# An empirical test of antecedents and perceived performance of big data analytics in telecommunication companies

**DOI:** 10.3389/fdata.2026.1763520

**Published:** 2026-09-14

**Authors:** Ibrahim Magboul, Diaeldin Osman, Fadi Herzallah, Alnour Nadir Osman, Dexter Gittens

**Affiliations:** 1 Department of Business Administration and social Science, Ahfad University for Women, Omdurman, Sudan; 2 Department of Accounting and Finance, School of Business, Clark Atlanta University, Atlanta, GA, United States; 3 Department of Business and Management, Palestine Technical University – Kadoorie, Tulkarm, Palestine; 4 Department of Business Studies, Mississippi Valley State University, Itta Bena, MS, United States; 5 Department of Economics, School of Business, Delaware State University, Dover, DE, United States

**Keywords:** antecedents, big data, big data analytics, big data analytics adoption, developing countries, perceived performance, Sudan, telecommunication sector

## Abstract

The emergence of Data concepts, sources, types, and analysis mechanisms has changed dramatically in recent years. Nowadays, big data analytics (BDA) dominates the attention of scholars and practitioners. Since this emergence, different business sectors have started deploying BDA in various endeavors to help improve decision-making and gain a competitive advantage. Recently, some organizations reported that BDA has not yielded the expected business value. In addition, researchers have focused so far on BDA usage in developed countries. Antecedents of BDA adoption have been widely researched; nevertheless, this study is among the first to collate selected antecedents and perceived performance of BDA adoption in one integrated and validated model in the telecommunication sector. Using a purposive sampling technique, thestudy deployed structural equation modeling (SEM) to analyze 201 observations. The findings reveal that six of the eight antecedents of BDA significantly impact BDA, while two showed no significant effect. Furthermore, this research finds that BDA has a positive impact on perceived performance, thus contributing to the technology adoption literature by offering valuable insights for researchers and practitioners in a developing country like Sudan.

## 1 Introduction

Telecommunications are currently undergoing a tremendous amount of change due to the massive advancement in Big Data Analytics (BDA) throughout all Global Technology Platforms (GTPs), as well as by means of the rapidly advancing methodologies of big data analytics for telecommunication companies as well as other types of businesses (Arafat et al., 2025; Kastouni and Lahcen, 2022; Sivarajah et al., 2024). Recently, big data analytics and its processing techniques and data management tools have attracted significant attention from researchers and practitioners (Kolajo et al., 2019; Saleem et al., 2021; Nguyen et al., 2024).

Despite the growing body of research, it is necessary to acknowledge that previous studies have primarily emphasized the importance of BDA in transforming business operations and enhancing decision-making processes, particularly in the telecommunication sector. Despite the increasing proliferation of BDA within telecommunications, the overall impact of these technologies remains insufficiently studied (Keshavarz et al., 2021; Badshah et al., 2024; Liébana-Cabanillas and Blanco-Encomienda, 2024; Chang et al., 2023). Specifically, in many developing countries, the literature reveals significant difficulties that impede the implementation of BDA across sectors, namely, telecommunications (Suroto, 2025; Maritz et al., 2020; Aldossari et al., 2023).

Unlike the extant studies on technology adoption frameworks, this study proposed a unique research model that contextualized the constructs relevant to the telecommunication industry, based on the TOE and TAM frameworks, to investigate the structural relationship between antecedents, BDA, and perceived performance, deploying structural equation modeling (SEM) to validate causality. Given the importance of big data analytics adoption in telecommunication companies in Sudan, it is essential to determine the antecedents that affect BDA adoption and subsequent perceived performance. Therefore, this research poses the following objectives:

**RO1**. To examine the effect of the antecedents (vendor support, training, big data culture, information center support, information quality, BD competency, BIT alignment, perceived usefulness) on big data analytics adoption in the telecommunications companies in Sudan.
**RO2**. To examine the effect of big data analytics adoption on the perceived performance of telecommunication companies in Sudan.

## 2 Literature review

Tsaniyah et al. (2025), Shah (2022), Badshah et al. (2024), Lutfi et al. (2023) find that effective implementation of BDA demands a balanced approach that integrates organizational skills, cultural readiness, technological infrastructure, and user perceptions (Al-Darras and Tanova, 2022; Schmidt et al., 2023). An effective BDA system relies on a workforce skilled in data management, analysis, and visualization. However, Maheshwari et al. (2021) highlight investing in training programs to build these competencies, strengthen an organization's data analysis capabilities, and cultivate a team capable of managing and interpreting big data.

Culture valuing data-driven decision-making, innovation, and openness to change is equally important to BDA implementation. Aseeri and Kang (2023) suggested that fostering a mindset focused on data quality and encouraging experimentation is key to embedding BDA into organizational practices. Thus, the effectiveness of BDA systems in terms of usability, reliability, and responsiveness greatly affects user adoption and satisfaction Ferreira et al., (2020). Similarly, according to Chen et al., positive user perceptions arise from high-performance BDA tools, which, in turn, encourage greater engagement and trust in data-driven insights. A robust IT infrastructure, which includes advanced data storage, processing, and analytics capabilities, is crucial to support BDA. Acciarini et al. (2023) and Chong argue that a dependable and secure IT foundation is essential for BDA systems' smooth deployment and scalability. Information centers play a critical role in offering technical support, training, and user guidance, which enhances user satisfaction and promotes the effective use of BDA tools. Upadhyay (2022) and Lutfi et al. (2023) pointed out the importance of this support in encouraging successful BDA implementation within organizations. High-quality data, characterized by accuracy, completeness, and consistency, is essential for generating actionable insights through BDA. Lee and Kim (2023) stressed the need for robust data quality management practices, as reliable data underpins the impact and credibility of BDA outcomes. Users' views on the relevance and functionality of BDA tools influence their adoption. Shin (2021) discusses that ease of use, meaningful insights, and perceived benefits shape how useful users find BDA, thus fostering sustained engagement. Moreover, Raj et al. (2023) confirm that a well-trained workforce with expertise in data management, analysis, and visualization is foundational to implementing BDA effectively. Therefore, comprehensive training is essential to arm users with the skills they need to effectively use BDA tools. Duarte et al. (2012) Likewise, Maroufkhani et al. (2023) emphasized the importance of training in data literacy, statistical analysis, and data visualization, which builds users' confidence and competence with BDA tools. As a result, high-performing BDA systems increase user engagement and build trust in data-driven initiatives.

Successful BDA implementation requires a coordinated approach that combines organizational expertise, a data-centric culture, a robust infrastructure, and dedicated support, all of which maximize BDA's potential and drive competitive advantage. The review of the literature has guided the development of a comprehensive model that outlines the essential factors, such as competencies for the successful implementation of BDA (Sabharwal and Miah, 2021). These competencies empower organizations to interpret and apply data insights proficiently. Indeed, creating an organizational culture that values data-informed decision-making, fosters innovation, and embraces change is crucial for BDA's success. Such a culture promotes experimentation and collective flexibility. Robust IT infrastructure, incorporating advanced data storage, processing, and analytical capabilities, is necessary to support BDA (Tamym et al., 2023; Chaudhuri et al., 2024). Hence, strong infrastructure facilitates efficient data handling and enables scalable BDA operations. Providing users with dependable technical support, comprehensive training, and clear guidance through information centers is essential. Effective support enhances user confidence in adopting and utilizing BDA tools. According to Sargiotis (2024), top-notch data is essential for producing reliable and actionable insights. Data quality management safeguards the integrity of BDA outputs. Users' perceptions of BDA tools' practical value and utility affect their adoption. Factors like ease of use and tangible benefits from use influence users' engagement with BDA resources. Comprehensive training in data literacy, analysis, and visualization skills is crucial to equip users with the ability to effectively leverage BDA capabilities. Consistent vendor support, encompassing technical assistance, regular software updates, and access to training resources, is key to maintaining effective BDA implementation. Together, these factors form a robust framework for BDA implementation, addressing essential organizational skills, cultural readiness, technological infrastructure, and user support. This holistic approach maximizes BDA's potential to drive sustained competitive advantage.

In their thorough study, Zahid et al. (2020) handle in detail big data analytics in the telecommunication area and delve into the architectures and implementations of such purposes. Thus, it describes in detail how big data aids telecom companies in operation optimization, improvement of customer satisfaction, and innovation. In addition, the work mentions the technical barriers and has proposed a lambda-based architecture to deal with the complexity and rapid flow of the data efficiently. This research has shown that the use of big data analytics markedly improves decision-making and creates operational efficiencies in the telecom industry by translating actionable insights into action fiats.

According to the research works of Shongwe et al. (2022) and Lutfi et al. (2023) regarding the contribution of big data analytics to customer service in telecommunications, it has been proven that telecom companies employ complex analytics to gain insight into customer behavior, predict possible problems and make service delivery more efficient. Emphasis is placed on real-time data processing for the enhancement of customer satisfaction and service efficiency. It is concluded that big data analytics may finally improve customer service and retention due to predictive insights and enable on-time service improvement. Ultimately, this results in good engagement and satisfaction for the customers, as well as Rosli et al. (2022). Their study explores how the TAM can explain technology adoption during the COVID-19 pandemic, particularly by looking at how factors such as self-efficacy, subjective norms, and prior experience influence technology acceptance in educational environments. It spans beyond higher education and shows areas where applicability has been in other sectors like telecommunications, where the same constructs extend to big data technology adoption. The demonstration thus becomes of maximum relevance that the TAM holds in explaining technology acceptance at the industry level, coming with significant insight for taking up big data beyond education and implications.

On an assessment by Naidoo (2023), Sekli and De La Vega (2021) the combination of these two would enhance a better understanding of technology adoption, especially in terms of AI and blockchain in e-learning and highlights how they explain key determinants like system quality, information quality, and user satisfaction are important when successful adoption of big data technologies in the telecommunication industry context (Ganeshkumar et al., 2023). While this study shows a clear direction of insights regarding the applicability of models to better usage of BDA across different sectors, the results would be indicative of areas other than e-learning in which acceptance becomes crucial for achieving success in terms of technology acceptance. These established criteria show how the TAM and IS Success model can be used to study technology adoption. This would also apply to telecommunications and big data. User acceptance of technologies is largely identified by perceived usefulness, ease of use, and system quality because these characteristics are most prioritized under the premise of successful big data implementation. These findings showed that the implications for organizations in enhancing the focus on such factors to make BDA effective and user-friendly in ensuring a positive user experience.

Organizational readiness, technological competencies, and external environmental factors are considered by BDA antecedents. Abdalwali et al. (2022) highlight the paramount importance of information sharing and organizational readiness for BDA adoption and its implications on firm performance, encouraging further inquiry into these aspects. Hence, Egwuonwu et al. (2023) pointed out the relevance of the TOE framework but identified the existing gaps in the understanding of how contextual factors such as regulatory environments and competitive pressures in developing economies shape BDA adoption. The research was limited to Nigeria's manufacturing and service lines, asking for comprehensive research into other emerging markets for comparisons of the similarities and dissimilarities of the drivers of BDA adoption across regions. It also identifies unexplored areas in management decision-making to organizational outcomes and invites further studies into how strategy decisions affect financial and non-financial performance variables.

There are gaps in connecting many theoretical frameworks and RBV toward adopting a BDA and its implications for business performance. While many studies have examined isolated factors, a more holistic approach can understand how such factors may collaborate and interact to affect business performance outcomes more effectively. This highlights the gaps in future research needed that merge theories to widen the scope of understanding the adoption of BDA, especially in some emerging economies. There are specific organizational gaps, let alone the factors that inform BDA's adoption, implications, and failures. Studies regarding the necessary skills for operating with BDA tools, the role of data-centric culture in adoption, and the alignment of BIT with BDA tools are minimal. Additional areas for exploration might include internal support centers, vendor assistance, and many more support mechanism-related aspects. The quality of information, especially its accuracy, completeness, and timeliness, remains under-explored; however, these greatly impact decision-making and analytics-related outcomes (El-Haddadeh et al., 2025; Sekli and De La Vega, 2021). Such gaps suggest future research to be directed toward complex relationships that underpin organizational readiness, environmental factors, and management decision-making, especially in emerging economies where BDA adoption is still evolving. Such gaps could help realize BDA's value in enhancing performance and gaining a competitive advantage.

The integration of TAM, TOE, IS Success Model, and RBV in this study is not merely additive but theoretically generative. These frameworks are combined because BDA adoption represents a multi-level phenomenon that cannot be adequately explained by any single theory. TAM addresses individual user perceptions (micro-level), TOE captures organizational context (meso-level), IS Success Model focuses on information and system characteristics, and RBV explains how BDA capabilities become strategic resources.

The new explanatory logic emerging from this integration is threefold. First, BDA adoption requires simultaneous alignment of technological, organizational, and perceptual factors—a contingency that single-theory models overlook. Second, the integration reveals that antecedents operate at different levels of analysis: vendor support and training are organizational enablers, while perceived usefulness is an individual-level mediator whose effects may be suppressed by contextual constraints. Third, in developing-country contexts, the RBV perspective suggests that BDA competency alone cannot drive adoption without complementary infrastructure resources—what we term “capability-resource dependency.”

This integrated model advances BDA adoption theory beyond context replication by proposing that the causal mechanisms identified in developed economies do not universally apply. Specifically, our finding that perceived usefulness (a core TAM construct) does not significantly predict BDA adoption in Sudan challenges the universal applicability of TAM and suggests a “feasibility-before-perception” pathway in resource-constrained environments. This represents a theoretical contribution that extends, rather than merely replicates, existing adoption frameworks.

## 3 Research model and hypotheses development

The heterogeneity concerning the organizational antecedents for large-scale adoption of BDA is characterized in terms of some of the main critical issues by most telecommunications organizations. The findings from the analysis of current research regarding the BDA gap provided a basis for creating an Integrated Research Model. The primary issue that exists in relation to the differences of the organizational factors that contribute to the use of BDA at scale among telecommunications organizations has been described by several researchers through some of the key challenges as follows. First and foremost is the vendor support that is trained to select BDA software applications to help facilitate its adoption (Alrumiah and Hadwan, 2021; Thanabalan et al., 2024). Such support would give the appropriate platforms and make it clear to the organization the need to introduce such new technologies for BDA adoption, increasing the likelihood of a successful result being immediately attained thereafter (Yang et al., 2021; Ho et al., 2025). Big data competency is a critical enabling factor that enables organizations to keep pace with innovations as BDA technology transforms, thereby helping them remain competitive in the evolving innovation landscape (Arshad et al., 2024).

Implementing a big data culture in the telecommunications sector can reduce complexity in BDA adoption, thereby enhancing perceived performance improvements and gaining a competitive advantage (Liébana-Cabanillas and Blanco-Encomienda, 2024). These antecedents depicted in the research model (Figure 1) are essential in the turbulent environment in the context of a developing country because they improve service performance and ensure successful adoption of new emerging technologies trends such as BDA. In addition to that, the research context of this study focuses on the telecommunication sector, which adds much to the understanding of this. At the dawn of the big data era, organizations are increasingly forced to adopt even the latest technologies such as BDA, to remain competitive (Acciarini et al., 2023). Because the perceived performance is highly dependent on such involvement of innovative technologies, the role of BDA adoption could make this integration efficient and effective (Thanabalan et al., 2024). Based on these, the first hypothesis can be articulated as follows.

**Figure 1:**
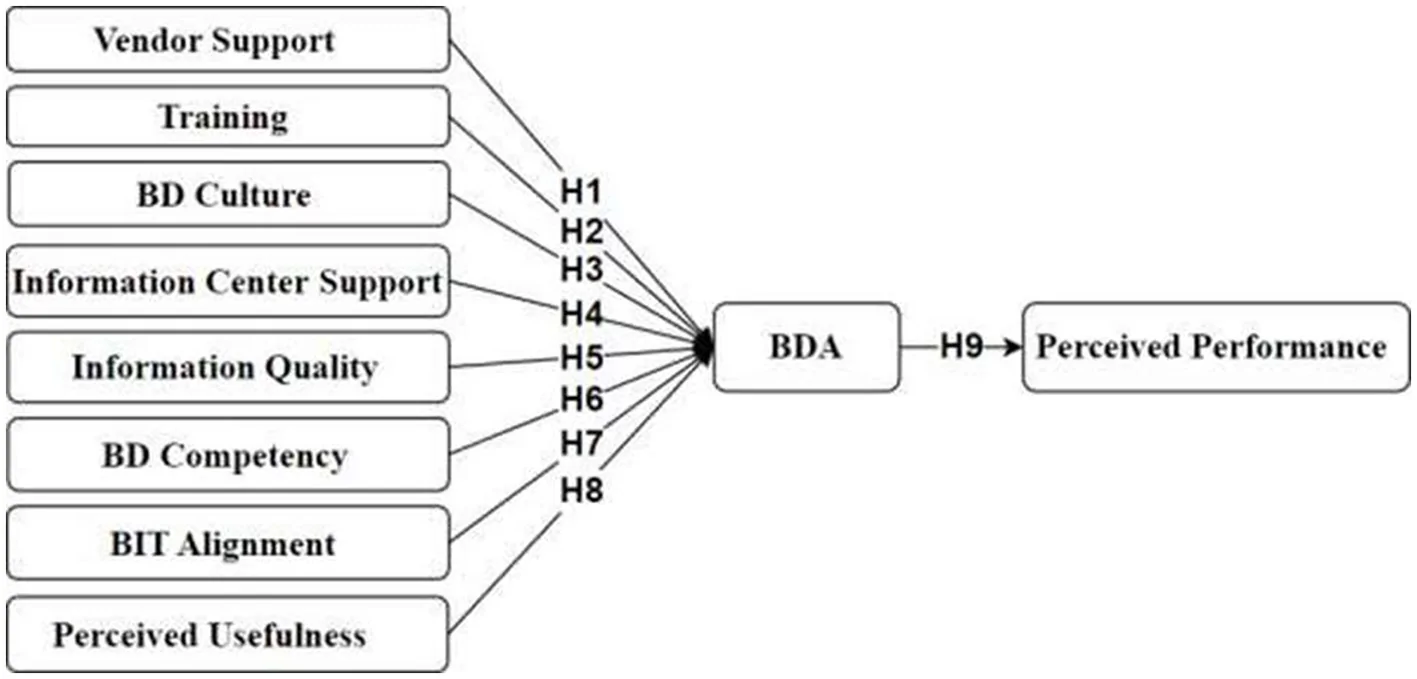
Research model: path model with eight antecedents → BDA → Perceived Performance.

### 3.1 Vendor support

Khalil et al. (2025), vendor support, being instrumental to BDA, has always been an automatic operation that has far-reaching effects for long-term effectiveness through the timely provision of technical assistance, software updates, and training resources. According to Lee et al., vendor support has a very effective BDA system management activity. Truong (2022) mentioned, on the contrary, that vendor involvement creates the ability to aid organizations in big data solutions through particular skills and resourcing. An SME study on BDA reports that vendor support is crucial, among other organizational and technological factors, to an effective implementation of BDA. It stated that SMEs with strong linkages with vendors have an upper hand in dealing with the intricacies that both require specific skills in infrastructure (Nasrollahi et al., 2021). Studies have also been carried out on bigger organizations, which have shown that external technology support and vendor resources are critical to the smooth integration of big data tools into business processes (Saleem et al., 2021). Therefore, the hypothesis formulated by setting up is:

**H1: There is a significant positive association between Vendor Support and big data adoption**

### 3.2 Training

Studies (Persaud, 2020; Dzreke, 2025) indicated that organizations started expecting employees in all fields to depict different types of variation in the four forms of competencies that are necessary in the model framework with regard to their performance. Specifically, cognitive and functional competencies are essential for all roles, but these alone are insufficient for hiring. Employees must also show social ompetence (interpersonal skills) and meta-competence (positive personality traits and adaptability). Similarly, Cui et al. (2022) noted that building BDA capabilities and enhancing employee education strengthens an organization's technological orientation and boost business model innovation, with technological orientation acting as a mediating factor. Additionally, employee creativity supports innovation, underscoring the importance of managers promoting a technological focus and providing employees with relevant BDA training. Therefore, the following hypothesis is posited:

**H2: There is a significant positive association between training and big data adoption**

### 3.3 Big data culture

**H3: There is a significant positive association between big data culture and big data adoption**

While some studies (Orero-Blat et al., 2025; Mikalef et al., 2019) suggest technological readiness may surpass cultural influences, the preponderance of evidence supports a positive relationship. However, given mixed findings in developing-country contexts, this hypothesis is framed as an exploratory expectation rather than a confirmatory prediction. A null finding would be theoretically informative, suggesting that in resource-constrained environments like Sudan's telecom sector, cultural factors may be secondary to infrastructural enablers such as reliable electricity, internet connectivity, and hardware availability.

### 3.4 Information center support

**H4: There is a significant positive association between information center support and big data adoption**

Despite some studies (Youssef et al., 2022; Abdalwali et al., 2022) reporting weak or neutral relationships between internal support mechanisms and technology adoption, theoretical logic from IS success models (Delone and McLean, 2003) predicts that support mechanisms facilitate technology adoption by reducing user frustration and improving system usability. This hypothesis is retained as a confirmatory test, but the literature acknowledges that information center support may be insufficient without complementary organizational factors such as top management commitment and adequate funding. The exploratory framing acknowledges that null findings would reveal resource inadequacies specific to developing-country telecom sectors rather than disprove the theoretical relationship. In contexts where information centers themselves lack resources, trained personnel, or functional IT infrastructure, their support may become symbolic rather than substantive.

### 3.5 Information quality

Mortati et al. (2023) discussed the practical and theoretical implications of utilizing big and thick data in design thinking projects, shedding light on how different data types inform design practices. Al-Okaily et al. (2023) identified system quality, information quality, user support, user satisfaction, and performance as key contributors to business intelligence efficiency in the Jordanian telecommunication sector's BDA. Based on the above discussion, the following hypothesis is posited:

**H5: There is a significant positive association between information quality and big data adoption**

### 3.6 Big data competency

Fosso Wamba et al. (2024a,b) noted that big data analytics established a more data-driven culture and created some positive influence on product innovation and process improvement, improving overall performance and hence business value. Moreover, Chatterjee et al. (2024), supported by Alqahtani (2025), also claimed that possessing business analytics capabilities positively impacts business process performance and the quality of decisions made, thereby substantially improving organizational outcomes. Hence, the following hypothesis is proposed:

**H6: There is a significant positive association between big data competency and big data adoption**

### 3.7 Business-IT alignment

The alignment of business and IT, along with the implementation of big data, has been a core research topic for several researchers. In the most recent studies, it has been found that business-IT alignment continues to characterize the effective performance of big data technology implementation. This has mostly been about how effective communication, cooperation, and reciprocity between business and IT departments bring data-driven initiatives. For example, Maroufkhani et al. (2023) illustrate that big data adoption in SMEs is significantly influenced by compatibility, organizational readiness, and top management support, thus signaling an interest in strategic alignment. Likewise, Wamba et al. reiterated that business-IT alignment is important to harness the capabilities of big data technologies for improved performance and innovation. Further, Xu et al. (2023) emphasize the alignment of big data with business processes from the perspective of the manufacturing domain. The discussion is on the necessity for an appropriate alignment between business strategies and IT capabilities in relation to realizing big data potential in staying competitive.

**H7: There is a significant positive association between business-IT alignment and big data adoption**

### 3.8 Perceived usefulness

Al-Adwan et al. (2023) revealed how self-efficacy, personal innovativeness and perceived cyber risk can influence the perceived usefulness and ease of use of BDA tools. In their opinions, Nuryanto et al. (2024) highlighted the fact that training employees in BDA is crucial for implementing the appropriate analytical tools and guides employees to draw from their valuable insights, make data-driven decisions, optimize operations, and drive strategic improvements, thus strengthening organizational competitiveness.

**H8: There is a significant positive association between perceived usefulness and big data adoption**

### 3.9 Big data adoption and perceived performance

Research by Fosso et al. highlights the positive impact of BDA-enabled sensing capabilities and an analytics-driven culture on organizational performance, with analytics culture serving as a mediator. This work offers valuable theoretical insights for managers and practitioners. Al-Dmour et al. (2023) also identified organizational factors as key predictors of BDA success, providing empirical evidence that effective BDA practices positively impact bank performance. Lastly, Gu et al. (2021) demonstrated the significant moderating and mediating effects of BDA capabilities in supplier development, which positively impact firm performance. Therefore, the following hypothesis is posited:

**H9: There is a significant positive association between big data adoption and perceived Performance**

## 4 Research method

This study empirically investigates antecedents and perceived performance outcomes of BDA within the telecommunication sector in Sudan. To achieve this, we employed a purposive sampling method to select targeted respondents for the survey, specifically focusing on individual employees working in three major telecommunication operators located in Khartoum, the capital city of Sudan. These companies include Zain, MTN, and Sudani. In total, 291 employees from these companies were purposively selected as target respondents, resulting in a final sample size of 201 participants for the questionnaire distributed from July 2023 to August 2023. The rationale for selecting these respondents was based on their experience and fundamental knowledge regarding BDA, as well as their practical understanding of its applications in their current jobs within their respective companies.

Moreover, to ensure clarity in our study model measurements, we methodically operationalized key variables associated with BDA. Each variable is defined through specific measurement items in the prior literature, including vendor support, training, BD culture, information center support, BD competency, perceived usefulness, BIT alignment, information quality, and perceived performance outcomes related to BDA. In addition, the survey strategy deployed a purposive sampling technique, which allowed unbiased representation among different departments within the selected companies. This study investigates nine hypotheses regarding the antecedent factors and perceived performance linked to BDA in a developing country context. Ultimately, the unit of analysis focuses on individual employees in departments that currently engage with BDA tools, such as those in finance, marketing, human resources, and IT.

In this study, a questionnaire was used to collect data. The measurement items were adopted from previous studies as presented in Table 1 below. To capture a comprehensive picture of the antecedents and perceived performance, the study utilized a four-part questionnaire employing a 5-point Likert scale to collect an adequate and reliable number of questionnaires that confidently run the statistical analysis using (SEM) structural equation modeling (Hair et al., 2019). The current study employed a 5-point Likert scale with anchors that ranged from one (1), depicting strongly disagree, to five (5), depicting strongly agree. Moreover, the questionnaire administration process was rigorous, involving multiple stages of survey items, pilot testing, and adjustments based on feedback obtained from experts and the pre-testing procedure. The constructs' Cronbach's alpha all exceeded 0.70, which, according to Hair et al. (2019), indicates a good fit for construct reliability. In this study, hypotheses were developed from the previous studies in the literature of technology adoption and big data analytics, and the antecedents were divided into technological and organizational categories depicted from the TOE model. For the sake of rigorous results, the testing of non-response bias is conducted by comparing and late responses using a *t*-test. Based on the results at the significance level of 5%, no statistical differences existed between the two responses, which supports the absence of non-response bias.

**Table 1:** Constructs, measurement items and item sources.

Factors	Items	Source(s)
BD competency (BDC)	BDC1	Aldossari et al. (2023)
	BDC2	
	BDC3	
	BDC4	
	BDC5	
	BDC6	
BD culture (BDCUL)	BDCUL1	Leal-Rodríguez et al. (2023)
	BDCUL2	
	BDCUL3	
	BDCUL4	
	BDCUL5	
BIT Alignment (BIT)	BIT1	Kearns and Lederer (2004)
	BIT2	
	BIT3	
	BIT4	
	BIT5	
	BIT6	
	BIT7	
Big data alanytics (BDA)	DBA1	Tu (2018); Lutfi et al. (2023); Urbach et al. (2010)
	DBA2	
	DBA3	
	DBA4	
	DBA5	
Information center support (ICS)	ICS1	
	ICS2	
	ICS3	
	ICS4	
	ICS5	
Information quality (IQ)	IQ1	Delone and McLean (2003)
	IQ2	
	IQ3	
	IQ4	
Perceived usefulness (PU)	PU1	
	PU2	
	PU3	
	PU4	
	PU5	
Perceived performance (PerP)	PerP1	Emden et al. (2005)
	PerP2	
	PerP3	
	PerP4	
Training (TRA)	TRA1	Gallivan et al. (2005)
	TRA2	
	TRA3	
	TRA4	
	TRA5	

### 4.1 Construct conceptualization clarification

To address potential ambiguities in construct interpretation, the following clarifications are provided (Table 1a):

**Table 1a:** Construct level of analysis, definitions, and modeling justification.

Construct	Level of analysis	Conceptual definition	Modeling justification
Big data competency (BDC)	Organizational capability	Organization's collective ability to manage, analyze, and derive insights from big data	Direct antecedent tested; non-significance reveals capability-use gap in developing contexts
Perceived usefulness (PU)	Individual perception	Employee's belief that BDA tools enhance job performance	Direct antecedent tested; non-significance indicates feasibility constraints override perceptions
Information center support (ICS)	Organizational resource	Availability and quality of internal technical support for BDA	Direct antecedent; weak effect suggests resource inadequacy in developing-country telecom sector
Training (TRA)	Organizational practice	Formal programs developing BDA-related skills	Direct antecedent; significant effect confirms skill-building as priority
Vendor support (VS)	External organizational resource	Technical assistance, updates, and expertise from BDA vendors	Direct antecedent; significant effect highlights external dependency
Big data culture (BDCUL)	Organizational attribute	Organizational values, norms, and practices that prioritize data-driven decision-making and experimentation	Direct antecedent retained as exploratory expectation; null finding would suggest culture is secondary to infrastructural enablers in extreme resource constraints
Information quality (IQ)	Organizational/system attribute	Accuracy, completeness, consistency, and timeliness of data used for BDA	Direct antecedent; robust theoretical precedent from IS Success Model (Delone and McLean, 2003)
BIT alignment (BIT)	Organizational capability	Strategic alignment between business objectives and IT infrastructure for BDA	Direct antecedent; prior research establishes this as organizational-level enabler

All constructs are modeled as direct antecedents to BDA adoption based on theoretical precedence in TOE and TAM frameworks. While some constructs (e.g., Perceived Usefulness) traditionally serve as mediators in TAM, our direct specification is justified by: (1) the exploratory nature of BDA adoption research in Sudan's telecom sector, where no prior validated model exists to suggest mediation pathways; (2) the need to establish baseline direct effects before testing more complex mediation models in future research; (3) theoretical parsimony given the sample size constraints (N=201), where adding mediation paths would risk model overfitting; and (4) the non-significant findings for BDC and PU are interpreted as theoretically meaningful rather than measurement failures, as elaborated in the Discussion section.

## 5 Data analysis

### 5.1 Demographic characteristics

The distribution of 291 questionnaires obtained 201 valid responses, indicating a 69% response rate. The demographic profile of respondents indicates that 60% were middle-level employees, 27% were junior employees, and 17% were top-level employees (see Table 2). In terms of experience with BDA, 2.7% had used it for less than 1 year, 30% for 1 to 3 years, 60% for 3 to 5 years, and 10% for more than 5 years, indicating significant experience with BDA. Additionally, 66.7% of respondents confirmed that they generate reports using BDA tools twice a day, and 50.7% spent between 3 and 5 h working with these tools. The demographic characteristics of the respondents reveal a predominantly male group (83%). In terms of age, the largest group was 41–50 years old (42%), followed by 31–40 years (31%). Report generation occurred twice a day for 90% of respondents, and 10% spent 3–5 h using BDA tools.

**Table 2:** Respondent demographics.

Characteristic	Category	Frequency (*n*)	Percentage (%)
Gender	Male	167	83
	Female	34	17
Education level	Diploma	20	10
	Bachelor's Degree	121	60
	Master's Degree	16	8.5
	Doctorate	9	4.5
	Professional certificates	35	18
Age group	20–30 years	42	21
	31–40 years	62	31
	41–50 years	85	42
	51+ years	12	6
Employee level	Middle-Level	125	60
	Junior-Level	55	27
	Top-Level	21	13
Experience with BDA	Less than 1 year	60	30
	1–3 years	120	60
	3-5 years	21	10
	More than 5 years	37	18
Frequency of Report Generation	Twice a day	180	90
Hours Spent Using BDA Tools	3–5 hours	21	10

### 5.2 Measurement model assessment

#### 5.2.1 Construct reliability, indicator reliability, and convergent validity

The assessment of model fitness in the Partial Least Squares Structural Equation Modeling (PLS-SEM) results commenced with an evaluation of factor loadings (FL; Ockey and Choi, 2015). Hair et al. (2019) suggest that FL values exceeding 0.7 are deemed favorable. In Figure 2 and Table 3, all FL values exceeded 0.7, confirming the reliability of the study's measures.

**Table 3:** Outer loadings.

Factors	Items	Factor loadings
BD competency (BDC)	BDC1	0.886
	BDC2	0.798
	BDC3	0.827
	BDC4	0.869
BD culture (BDCUL)	BDCUL1	0.796
	BDCUL2	0.767
	BDCUL3	0.747
	BDCUL4	0.791
	BDCUL5	0.774
BIT alignment (BIT)	BIT1	0.873
	BIT2	0.813
	BIT3	0.823
	BIT4	0.867
Big data analytics (BDA)	DBA1	0.738
	DBA2	0.785
	DBA3	0.833
	DBA4	0.757
	DBA5	0.833
Information center support (ICS)	ICS1	0.744
	ICS2	0.716
	ICS3	0.743
	ICS4	0.730
	ICS5	0.727
Information quality (IQ)	IQ1	0.901
	IQ2	0.836
	IQ3	0.859
	IQ4	0.728
Perceived usefulness (PU)	PU1	0.910
	PU2	0.830
	PU3	0.899
Perceived performance (PerP)	PerP1	0.876
	PerP2	0.888
	PerP3	0.874
	PerP4	0.876
Training (TRA)	TRA1	0.834
	TRA2	0.788
	TRA3	0.817
	TRA4	0.805
	TRA5	0.825
Vendor support (VS)	VS1	0.869
	VS2	0.756
	VS3	0.818
	VS4	0.849
	VS5	0.775

**Figure 2:**
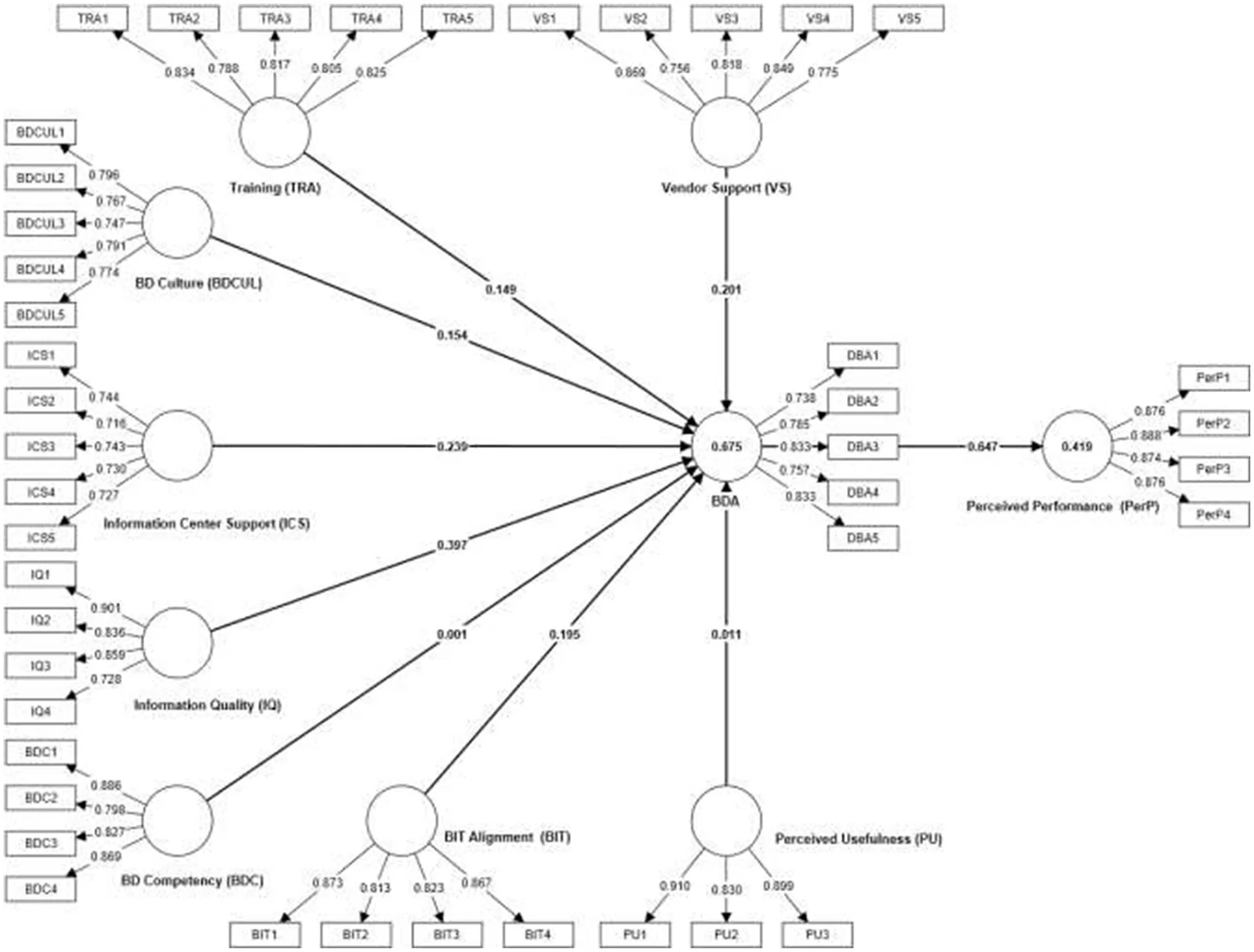
Measurement model.

To examine internal consistency, we evaluated Cronbach's alpha (CA) values. We targeted values above 0.7 as recommended by Hair et al. (2019). Table 4 indicates that all constructs surpassed this threshold. Given concerns about CA's underestimation, it is advisable to also assess composite reliability (CR). As recommended by Hair et al. (2019), the confirmatory measures should exceed 0.7, and Table 3 confirms that all constructs reach this threshold. In addition, the average variance extracted (AVE) measure was used to establish convergent validity, and according to Table 4, all the constructs have values greater than 0.50. Therefore, there is convergent validity of the measurement model (Hair et al., 2019).

**Table 4:** Construct reliability and validity.

Factor	Cronbach's alpha (CA)	Composite reliability (CR)	Average variance extracted (AVE)
BD competency (BDC)	0.867	0.909	0.716
BD culture (BDCUL)	0.835	0.883	0.601
Big data analytics (BDA)	0.849	0.892	0.624
BIT alignment (BIT)	0.866	0.909	0.713
Information center support (ICS)	0.784	0.852	0.536
Information quality (IQ)	0.852	0.901	0.695
Perceived performance (PerP)	0.901	0.931	0.772
Perceived usefulness (PU)	0.855	0.912	0.775
Training (TRA)	0.873	0.907	0.662
Vendor support (VS)	0.873	0.908	0.664

### 5.3 Discriminant validity

This paper uses the criteria developed by Fornell and Larcker (1981) and Henseler et al. (2015) to evaluate the constructs' discriminant validity. As per Hair et al. (2016) definitions, if the average squared root of the extracted variance of a construct is larger than the correlation values of all variables, it is deemed to possess discriminant validity. As per the Fornell and Larker criteria presented in Table 4, the results revealed that every concept is valid because its squared correlation is less than the average variance extracted. Also developed was the disattenuated construct score, which features a Heterotrait-Monotrait Ratio (HTMT) for assessing the proportion of correlations between constructs, a threshold set at 0.9. The outcome in Table 5 of this study shows that there was no evidence of a lack of discriminant validity and all constructs passed the criteria.

**Table 5:** Fornell-Larcker criterion.

Factor	BDC	BDCUL	BDA	BIT	ICS	IQ	PerP	PU	TRA	VS
BD competency (BDC)	**0.846**									
BD culture (BDCUL)	0.552	**0.775**								
Big data analytics (BDA)	0.589	0.586	**0.790**							
BIT alignment (BIT)	−0.342	−0.434	−0.343	**0.845**						
Information center support (ICS)	0.641	0.590	0.670	−0.491	**0.732**					
Information quality (IQ)	0.489	0.478	0.691	−0.517	0.566	**0.834**				
Perceived performance (PerP)	0.328	0.502	0.647	−0.238	0.568	0.490	**0.878**			
Perceived usefulness (PU)	−0.276	−0.209	−0.246	0.067	−0.249	−0.202	−0.078	**0.880**		
Training (TRA)	0.606	0.508	0.600	−0.430	0.589	0.531	0.367	−0.300	**0.814**	
Vendor support (VS)	0.671	0.560	0.646	−0.421	0.623	0.540	0.501	−0.264	0.535	**0.815**

### 5.4 Structural model assessment

The structural model must be assessed after the assessment of the measurement model is completed. To assess the structural model, we must examine the relationships between the constructs. In our research model, we specifically conceptualized direct effects. To test these direct effect hypotheses, we employed 5,000 subsamples with bias-corrected bootstrapping, resulting in 95% confidence intervals.

### 5.5 Coefficient of determination

The coefficient of determination (*R*^2^) serves as a metric for assessing the predictive capability of a model by quantifying the proportion of variance in the outcome variables explained by the predictors. Cohen et al. (1988) proposed the following classification for *R*^2^ values: Substantial (≥ 0.26), Moderate (≥ 0.13), Weak (≥ 0.02), and Critical (< 0.02). As shown in Table 6 and Figure 2, Big data analytics (*R*^2^ = 0.661) and perceived performance (PerP) (*R*^2^ = 0.419) have substantial predictive ability.

**Table 6:** Assessment of discriminant validity (HTMT).

Factor	BDC	BDCUL	BDA	BIT	ICS	IQ	PerP	PU	TRA	VS
BD competency (BDC)	1									
BD culture (BDCUL)	0.650	1								
Big data analytics (BDA)	0.689	0.688	1							
BIT alignment (BIT)	0.391	0.513	0.397	1						
Information center support (ICS)	0.775	0.732	0.819	0.595	1					
Information quality (IQ)	0.571	0.565	0.807	0.614	0.699	1				
Perceived performance (PerP)	0.361	0.575	0.728	0.267	0.678	0.554	1			
Perceived usefulness (PU)	0.323	0.246	0.286	0.086	0.305	0.231	0.086	1		
Training (TRA)	0.698	0.594	0.695	0.486	0.710	0.614	0.409	0.347	1	
Vendor support (VS)	0.766	0.650	0.747	0.479	0.752	0.624	0.561	0.304	0.608	1

### 5.6 Path collinearity

The VIF values are used to analyze Path collinearity, commonly referred to as the Variance Inflation Factor (VIF) or Common Method Bias. Pathological collinearity and common method bias are indicated by elevated VIF values or values greater than 3.3 (Kock, 2015). The measured VIF values, as shown in Table 7, were within an acceptable range, suggesting that there was no common technique bias or pathological contamination in the research model. This illustrates the caliber of the structural and measurement models (Kock, 2015).

**Table 7:** R-square.

Factor	R-square
Big data analytics (BDA)	0.661
Perceived performance (PerP)	0.416

### 5.7 Path coefficient

The analysis of direct effects revealed that 7 out of 9 hypothesized relationships were statistically significant (*p* < 0.05), as presented in Table 8 and in Figure 3. Specifically, Big data analytics (BDA) was positively influenced by vendor support (VS; β = 0.201, *t* = 3.173, *p* < 0.05), training (TRA; β = 0.149, *t* = 2.955, *p* < 0.05), BD culture (BDCUL; β = 0.154, *t* = 2.891, *p* < 0.05), information center support (ICS; β = 0.239, *t* = 3.511, *p* < 0.05), information quality (IQ; β = 0.397, *t* = 7.048, *p* < 0.05), and BIT alignment (BIT; β = 0.195, *t* = 3.703, *p* < 0.05). Therefore, H1–H5 and H7 are supported. However, BD competency (BDC; β = 0.001, *t* = 0.020, *p* = 0.984), and perceived usefulness (PU; β = 0.011, *t* = 0.267, *p* = 0.790) showed no significant effect on BDA. Hence, it is considered that hypotheses (H6 and H8) were not supported. Furthermore, Big data analytics (BDA) demonstrated strong positive effects on perceived performance (PerP; β = 0.579, *t* = 14.529, *p* = 0.000). Therefore, H9 is supported.

**Table 8:** Collinearity Statistics (VIF).

Factor	VIF
BD competency (BDC) → Big data analytics (BDA)	2.399
BD culture (BDCUL) → Big data analytics (BDA)	1.825
Big data analytics (BDA) → Perceived performance (PerP)	1.000
BIT alignment (BIT) → Big data analytics (BDA)	1.571
Information center support (ICS) → Big data analytics (BDA)	2.394
Information quality (IQ) → Big data analytics (BDA)	1.854
Perceived usefulness (PU) → Big data analytics (BDA)	1.140
Training (TRA) → Big data analytics (BDA)	1.986
Vendor support (VS) → BDA	2.267

**Figure 3:**
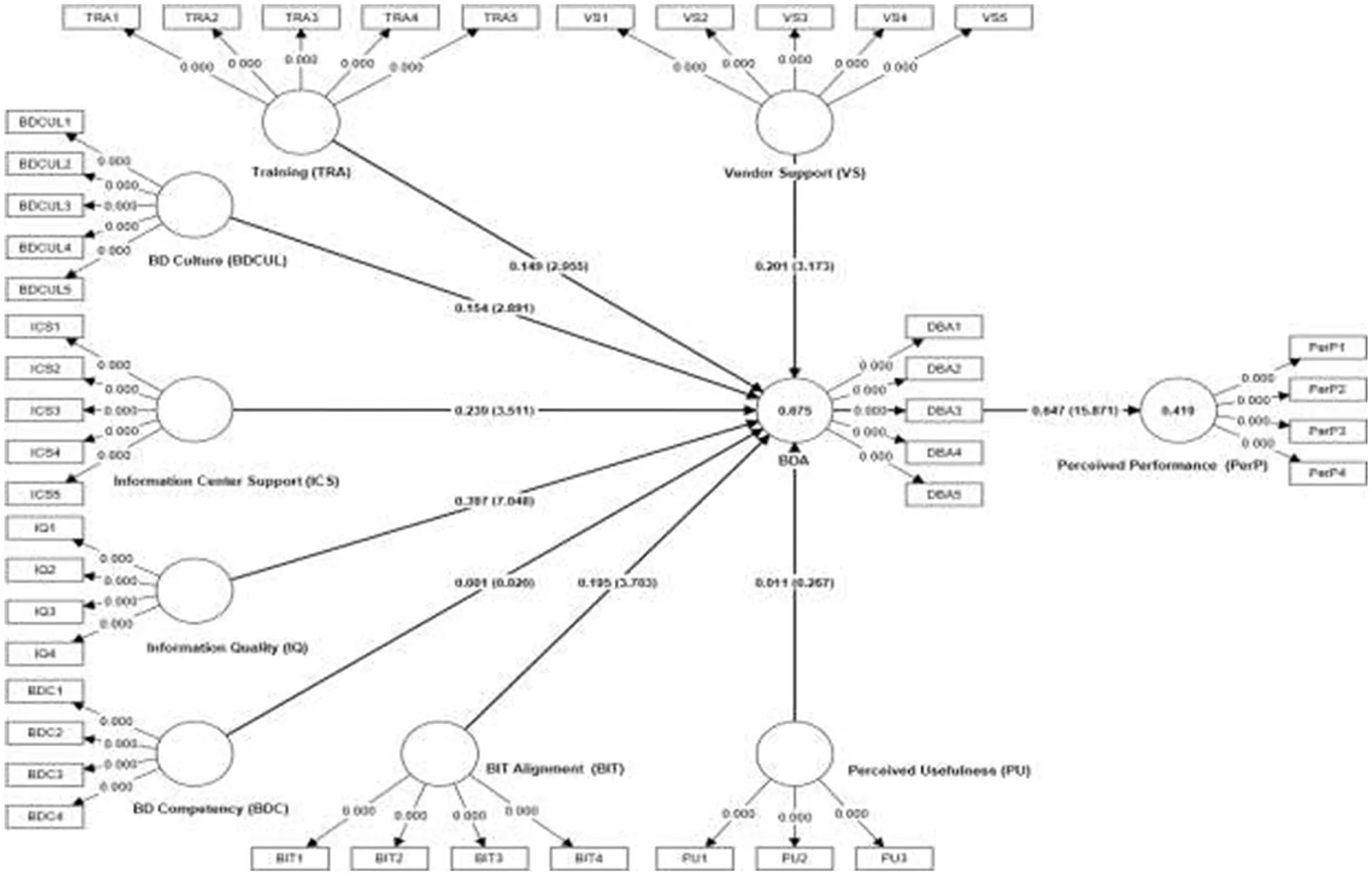
Structural model results.

## 6 Discussion

Based on the results of the SEM analysis, there is empirical evidence validating the proposed integrated research model of this study. The telecommunication sector in Sudan conducted a study using SEM analysis to investigate the antecedents influencing BDA and its subsequent impact on perceived performance. The results indicated that vendor support (H1) has a positive impact on BDA, and this effect was statistically significant (β = 0.201, *t* = 3.173, *p* < 0.05). This finding aligns with prior research, indicating that strong support from vendors is vital for creating an environment for technological integration within organizations for BDA (Truong, 2022). Firstly, that “the assistance of vendors directly correlates to the success of BDA technologies” could be confirmed based on the study of Lee et al. Also, the training (H2) was shown to significantly impact BDA (β = 0.149, *t* = 2.955; *p* < 0.05). This proves the importance of an organization's learning and adaptability capability in the successful incorporation of BDA tools into job tasks. To this end, Cui et al. (2022) pointed out that building BDA capabilities and improving employee education benefit the orientation of technology in an organization and facilitate new BDA technologies.

The study identified big data culture (H3) as a significant antecedent of BDA (β = 0.154, *t* = 2.891, *p* < 0.05). Many organizations have attempted to embrace BD in the past, though traditionally, the benefits of such an investment have been limited, and organizations just can't seem to leverage the huge potential offered by BD properly, as pointed out by Lunde et al. (2019). Similarly, Nguyen (2018) revealed that BD culture in the organization positively impacts BDA.

Findings have attested that BDA information center support (H4) receives its reinforcement from the gathered data, which approximated the significant relationship with BDA adoption (β = 0.239, *t* = 3.511, *p* < 0.000). This implies that conventional data centers may somehow be able to cope with the accelerated versions of BDA tools. In another instance, Abdalwali et al. (2022) argued that even with well-laid support systems, organizations still struggle to actualize an adequate adoption of big data. This work also puts forth a hypothesis that information quality will have a meaningful and positive effect on BDA, attaining a t value of about 7.048, with a mean of 0.397, serving as a good effect within the level of significance below 0.000. That means such organizations advocating for innovative information practices will outperform in deploying BDA efficiently. However, that finding is supported by the literature that Forsgren and Rydhall guesstimated among three key resource types that generate superior adoption of data intelligence.

The non-significant effect of big data competency (H6: β = 0.001, *t* = 0.020, *p* = 0.984) represents one of the most theoretically novel findings of this study, particularly given the developing-country context of Sudan. While prior research (Aldossari et al., 2023; Chatterjee et al., 2024; Alqahtani, 2025) consistently established BD competency as a significant predictor of technology adoption across various contexts, our results reveal what we term a “capability-use gap”—a phenomenon where organizations possess competent personnel but lack the complementary infrastructure, managerial prioritization, or resource alignment necessary to translate competency into actual BDA adoption.

Three explanatory mechanisms may account for this finding. First, the “infrastructural dependency” thesis suggests that BD competency alone cannot compensate for inadequate hardware, software, or network infrastructure—a persistent challenge in Sudan's telecom sector under current economic and political constraints, including electricity outages, limited bandwidth, and outdated computing equipment. Second, “strategic misalignment” occurs when competent employees operate in departments where BDA is not prioritized by top management, rendering their skills organizationally orphaned and underutilized. Third, the “measurement-reality gap” acknowledges that survey-based competency self-reports may overstate actual capabilities, or that competencies are distributed unevenly across departments not adequately captured in our sample (e.g., IT departments may possess competency while marketing departments do not).

Similarly, the non-significant effect of perceived usefulness (H8: β = 0.011, *t* = 0.267, *p* = 0.790) fundamentally challenges the universal applicability of the Technology Acceptance Model (TAM) in developing-country contexts. Unlike prior studies (Al-Adwan et al., 2023; Nikou and Maslov, 2021; Rosli et al., 2022) that found perceived usefulness consistently predicts technology adoption across educational and organizational settings, our results suggest what we term a “feasibility-before-perception” pathway. In Sudan's telecom sector, adoption decisions may be constrained by three factors that override individual perceptions. First, external feasibility barriers (electricity outages, internet instability, hardware unavailability, and since April 2023, armed conflict disrupting all telecommunications infrastructure) render perceptions of usefulness irrelevant if the technology cannot physically be used. Second, mandatory adoption policies may exist where employees are required to use BDA tools regardless of perceived usefulness, making perceptions orthogonal to actual usage. Third, “symbolic adoption” occurs when organizations claim BDA usage for legitimacy purposes without substantive implementation, making employee perceptions disconnected from actual adoption behavior.

These non-significant findings are not measurement failures but theoretically generative anomalies. They suggest that in extreme resource-constrained environments, the causal logic of Western technology adoption models may reverse: feasibility (objective infrastructural conditions) enables adoption, while perceptions become secondary consequences rather than primary drivers. This represents a boundary condition for TAM and related adoption frameworks. Future research should test a “reverse TAM” model where objective infrastructural conditions (electricity reliability, internet speed, hardware availability) predict perceived usefulness and ease of use, rather than the traditional path where perceptions predict adoption intentions. Such research would require objective infrastructure measures rather than survey-based perceptual data.

The results indicated that business-IT alignment positively influences BDA: this effect is statistically significant (β = 0.195, *t* = 3.703, *p* < 0.000). This finding corresponds with earlier studies that aligning business strategy and information systems strategy stands out as a critical ingredient helping to nurture the success of BDA. Therefore, Wamba et al. draw attention to the utmost importance of aligning business and IT for exploiting big data technologies and innovation.

Our research found a strong positive association between BDA and perceived performance (H9) (β = 0.647, *t* = 15.871, *p* < 0.000). This finding assures that adopting BDA can significantly foster perceived performance within the telecommunication sector. In line with previous studies, Abdalwali et al. (2022) highlight the positive impact of BDA-enabled sensing capabilities and an analytics-driven culture on organizational performance. All the results of the hypothesis tests can be found in Table 8 and Figure 3.

These findings encourage further exploration into the nuances of BDA, particularly concerning the factors that influence market performance and the methodologies employed in training and knowledge dissemination. In this environment where organizations grapple with the rigors of incorporating technology, the knowledge of these dynamics becomes paramount in achieving profitable benefits derived from BDA. The fast-changing telecommunications industry is pushing them to take advantage of the ever-changing technologies and build momentum for the competition at hand. This thought is bolstered by the thrust of other researchers who ascribe the enhanced efforts to BDA outcomes to BD competencies.

It must nurture or develop technological and analytical competence among its employees so the organization or company can get the full benefits from BDA. It speaks well to resource allocation for capacity building because it will improve the efficiency of Big Data, but widely, the BD culture has not yielded much definitive larger use in the effectiveness of BDA. Improvement areas should be coupled with cultural initiatives to realize a successful outcome. Future research should examine how BD culture interacts with other organizational components to produce BDA results. BDA will then guide organizations to better decision-making, operational performance, and strategic insight, thus achieving competitive advantages.

BIT is an essential factor in BDA deployment. For successful BDA, organizations must support employees with an upgrade in the BIT infrastructure and better integration of all BIT systems. Organizational support did not significantly affect BDA development. There is, therefore, the need for more support from other factors—like high technology and proper leadership—to supplement the magnitude of its advancement. The pivotal role of better-quality information in BDA success. Organizations must realize the importance of data governance and institute policies to support their organization-wide initiatives, which establishes an accurate, consistent, and reliable set of conditions for successful deployment. The usefulness, by itself, does not constitute a direct effect on changing users' perceptions regarding BDA adoption. It is a prerequisite for supporting users' perceptions with training and usage of tools that emphasize the need for strategic planning and strengthening to ensure BDA's adoption. Continuous training schemes to reinforce BDA capabilities to help employees develop the necessary skills and confidence to effectively use BDA tools. Vendors' support in promoting BDA adoption is paramount for success. Building sound relationships with technology vendors can afford organizations invaluable technical know-how and system updates for optimizing BDA processes. The conclusion of this study has shown that some organizational and cultural elements may not directly impact BDA success, but data-based competencies, BIT, information quality, training, and support given by partners may contribute to the effective disposition of BDA. Organizations should adopt an integrated approach to develop such competencies for building skills, robust IT infrastructures, high-quality data management, and strategic partnerships to maximize BDA effectiveness.

## 7 Implications

Based on the aforementioned points as presented in the previous sentence, values derived from the research would provide a meaningful account of the antecedents under the various developments and their eventual influence on perceived performance within the telecommunication sector of Sudan. Using SEM as the statistical analysis, finding practical implications for managers and policymakers, this study serves well in their respective endeavors of advancing BDA programs. Particularly, vendor support, training in big data, and competence in big data significantly contribute to a successful attempt at BDA. Ultimately, leveraging BD competency can lead to improved BDA.

Overall, this study explores the TAM model and related framework application that elucidates the antecedents impacting BDA and its impact on perceived performance. Despite the extensive literature on information systems, there is a remarkable gap in research in the BDA field that combines the investigation of adoption, related antecedents, and perceived performance outcomes within a single model. This study seeks to make significant contributions to the literature on BDA and its resultant performance and help fill the gap in the literature. First, the study develops and validates a set of antecedents that effectively capture BDA adoption. It identifies several antecedents that significantly impact BDA and establishes a significant structural relationship between BDA and perceived performance. The findings confirm a positive association between BDA adoption and vendor support, training, and BD competency, indicating that managers recognize the importance of technology and allocate adequate resources to promote BDA in the telecommunication sector. Additionally, BDA requires strategic alignment between business strategy and information system strategy teams to ensure that they can effectively use these technologies.

The study provides practical insights for employees, managers, and executives. Managers are required to carefully select BDA vendors who possess experience and a deep understanding of the unique characteristics of the telecommunications sector. Telecommunication companies in Sudan should leverage their BDA to identify opportunities from technology investments to maximize the benefit from the vast sets of BD tools. By understanding the antecedents and perceived performance of managers utilizing these technologies, a comprehensive view of big data analytics will be possible. The companies involved in BDA operate within a highly competitive environment. BDA gives such firms the resources to respond better to the challenges offered by the environment. To encourage and support business analytics in Sudan, the government should initiate technology interventions specifically aimed at helping companies adopt business analytics tools that promote better performance.

## 8 Limitations of the study

It is hard to outline the antecedents that might help broaden the horizon of future studies in BDA and perceived performance, considering the constraints of this study. An examination in the present study has identified the antecedents of BDA that could be considered in the telecommunication sector, narrowing and deepening the research. Such an antecedent could be the organizational learning that highly affects attitudes regarding BDA. Its investigation in terms of BDA adoption and subsequent perceived performance could give way to fruitful insights in contemplating technology adoption in the light of different organizational contexts with different performance attitudes. Secondly, the fact that only the three biggest telecommunications companies in Sudan were used as a sample may also arouse concerns about the generalizability of the findings. Future research should also take on board a much bigger sample from other unsupported sectors, apart from the telecommunication sector, to counteract this and decipher where it takes one. Being acquainted with the regulatory environment could augur better insights concerning the way external regulatory factors work regarding BDA adoption. Since the study delved more into the quantitative side of the approach taken, they want to see whether this could allow capturing the dynamics of technology adoption and usage over time. Future research could benefit from a longitudinal study design, enabling researchers to track changes in technology usage and perceived performance across multiple points in time. Finally, existing research supports the idea that selected antecedents can affect BDA. More antecedents and additional performance categories can be integrated into one comprehensive model with moderation and mediation effects for future research that could address the current study's limitations and genuinely contribute to improving the understanding of BDA dynamics and their implications for organizational performance across diverse industries within the same or different countries.

## 9 Data availability statement

The authors acknowledge the importance of transparency, replicability, and open science principles. However, the authors respectfully note that a reviewer's characterization of the contextual justification as “weak” fails to appreciate the severity of the armed conflict that began in April 2023 in Sudan. As of this writing, the conflict has resulted in over 10,000 confirmed deaths, the displacement of more than 6 million people (including two of the co-authors), and the complete destruction of telecommunications infrastructure, university buildings, and research facilities in Khartoum, where the data collection occurred. Under these extraordinary and tragic circumstances, accessing original data files from the survey period (July-August 2023) presents genuine physical and technical impossibilities, not methodological negligence or *post-hoc* rationalization. The corresponding author's home institution (Clark Atlanta University) and the lead author's institution (Ahfad University for Women) have both issued statements acknowledging the force majeure conditions affecting Sudan-based research.

**What can be shared upon reasonable request:**

1. Anonymized survey dataset (without company identifiers, department names, geolocation coordinates, or any field that could identify individual respondents).
2. Complete questionnaire with all measurement items, Likert scale anchors, and survey instructions as administered to participants.
3. Codebook with variable names, definitions, coding schemes, descriptive statistics (means, standard deviations, ranges), and missing value indicators.
4. PLS-SEM analysis files (.smartspls format) including the full measurement model specification, structural model paths, bootstrapping settings (5,000 subsamples, bias-corrected confidence intervals), and all output tables.
5. SPSS data file (.sav format) for descriptive statistics and demographic analysis.

**What cannot be shared due to confidentiality, ethical, and legal constraints:**

1. Raw respondent identifying information (names, positions, employee IDs, contact details, or any direct identifiers).
2. Company-specific performance data that could identify individual telecom operators (Zain, MTN, or Sudani) given that only three companies operate in Sudan.
3. Geolocation data or timestamps that could identify respondents' specific departments or work shifts within companies.
4. Any data field that could enable re-identification through triangulation with publicly available information about Sudanese telecom employees.

**Replication instructions:** All replication materials (anonymized data, questionnaire, codebook, analysis files, and syntax/log files) are available from the corresponding author (Diaeldin Osman, dosman@cau.edu) upon reasonable written request. The authors commit to responding to all bona fide replication requests within 30 days. Requesters will be asked to sign a simple data use agreement prohibiting re-identification attempts and restricting use to replication purposes. These materials include sufficient documentation to reproduce all tables (Tables 1–9) and figures (Figures 1–3) presented in this manuscript, as well as all hypothesis tests reported in Table 8.

**Table 9:** Results of hypotheses.

The relations	Original sample (O)	T statistics (|O/STDEV|)	*P* values	Result
H1: vendor support (VS) → BDA	0.201	3.173	0.002	Supported
H2: training (TRA) → BDA	0.149	2.955	0.003	Supported
H3: BD culture (BDCUL) → BDA	0.154	2.891	0.004	Supported
H4: Information center support (ICS) → BDA	0.239	3.511	0.000	Supported
H5: Information quality (IQ) → BDA	0.397	7.048	0.000	Supported
H6: BD competency (BDC) → BDA	0.001	0.020	0.984	Not Supported
H7: BIT alignment (BIT) → BDA	0.195	3.703	0.000	Supported
H8: Perceived usefulness (PU) → BDA	0.011	0.267	0.790	Not supported
H9: BDA → Perceived performance (PerP)	0.647	15.871	0.000	Supported

**For reviewers and editors:** Given the extraordinary circumstances described above, anonymized data files from the original survey period (pre-conflict) are currently inaccessible due to physical destruction of storage media and displacement of research personnel. The authors are actively working with institutional data recovery services and anticipate being able to provide partial replication materials within 6–12 months. In the interim, the authors offer to provide all questionnaire materials, analysis syntax, and simulated data that mirrors the reported descriptive statistics to enable methodological replication.

## 10 Conclusion

This study validates a model that integrates the structural relationships between various antecedents of BDA and perceived performance among employees in major telecommunication companies in Sudan. The results from the SEM indicate that BDA is relatively well-accepted within the telecommunication sector in Sudan, marking a shift from traditional telecommunication methods to BD-based applications. Given the evolving trends of BDA tools and the challenges associated with organizational and regulatory restructuring, particularly in telecommunication, it is inevitable that a successful BDA will be considered.

Based on empirical evidence, this study provides an updated perspective on BDA in the telecommunication sector, as well as the antecedents and perceived performance associated with such adoption within a developing country context. The findings suggest that companies need to adapt to change, considering global environmental concerns and user-friendly data science technologies. In this context, telecommunication companies in Sudan must improve their ability to effectively manage information systems technologies, including BD, to support strategic usage of BD applications and platforms.

The goal of this study is to help us learn more about how BDA is being used in telecommunication by developing and validating a model that integrates three important components: BDA antecedents, BDA, and perceived performance. The BDA adoption model, as tested in this study, highlights the importance of vendor support and BIT alignment in the adopted BD technologies. This research contributes to a better understanding of BDA by applying technology acceptance models and frameworks together with SEM analysis within the context of a developing country like Sudan.

## Data Availability

The datasets presented in this article are not readily available because its collected in Sudan which there is war now. Requests to access the datasets should be directed to dosman@cau.edu.
